# CELF1 represses *Doublesex1* expression via its 5’ UTR in the crustacean *Daphnia magna*

**DOI:** 10.1371/journal.pone.0275526

**Published:** 2022-10-14

**Authors:** Yusrifar Kharisma Tirta, Shungo Adachi, Christelle Alexa Garcia Perez, Nikko Adhitama, Quang Dang Nong, Toru Natsume, Yasuhiko Kato, Hajime Watanabe

**Affiliations:** 1 Department of Biotechnology, Graduate School of Engineering, Osaka University, Suita, Japan; 2 Cellular and Molecular Biotechnology Research Institute (CMB), National Institute of Advanced Industrial Science and Technology (AIST), Tokyo, Japan; 3 Institute for Open and Transdisciplinary Research Initiatives (OTRI), Osaka Univeristy, Suita, Japan; Leibniz Institute on aging - Fritz Lipmann Institute (FLI), GERMANY

## Abstract

In sex determination of the crustacean *Daphnia magna*, male-specific expression of DM-domain transcription factor Doublesex1 (Dsx1) orchestrates the male developmental program triggered by environmental stimuli. We previously identified the CELF1 ortholog as a candidate of proteins associated with the 5’ UTR of the *Dsx1α* isoform. Here we report the CELF1-dependent suppression of *Dsx1* expression in *D*. *magna*. During embryogenesis, *CELF1* expression was not sexually dimorphic. Silencing of CELF1 led to the activation of *Dsx1* expression both in female and male embryos. Overexpression of CELF1 in male embryos resulted in a reduction of *Dsx1* expression. By these manipulations of CELF1 expression, the *Dsx1* transcript level was not significantly changed. To investigate whether the CELF1 controls Dsx1 expression via its 5’ UTR, we injected the GFP reporter mRNA having intact *Dsx1α* 5’ UTR or mutated one lacking the GU-rich element (GRE) that is known as a binding site of the CELF1 ortholog. We found that deletion of the GRE significantly increased the reporter gene expression. These results indicate that CELF1 suppresses Dsx1 expression both in females and males, possibly at the post-transcriptional level. We speculate that CELF1 may avoid unintended Dsx1 expression and generation of sexual ambiguity by setting a threshold of Dsx1 expression.

## Introduction

In favourable conditions, the freshwater crustacean *Daphnia magna* produces only females by parthenogenesis. In contrast, under a stressed environment such as shortened photoperiod, a lack of food, and/or increased population density, *D*. *magna* produces male offspring that are genetically identical to females [1, 2]. The environmental cues for male determination stimulate the neuroendocrine system and secrete sesquiterpenoid, which promotes the production of parthenogenetic eggs that are destined to develop into males [3, 4]. The male developmental program is implemented by the male-specific expression of the DM-domain containing transcription factor named Doublesex1 (Dsx1) [5]. Ectopic expression of Dsx1 in females could lead to sexual ambiguity [5, 6]. Since the intersex of *Daphnia* is rare in nature, there would be robust regulation of the endogenous Dsx1 expression. The *Dsx1* gene produces two isoforms, α and β, which differ only in the 5’ UTR [5]. Previously, we investigated the proteins bound to the 5’ UTR of the *Dsx1α* isoform and identified CUGBP1 protein as one of the associated protein candidates [7]. However, its role in the *Dsx1* regulation remains unknown.

CUGBP1 is an RNA-binding protein harboring three RNA recognition motifs (RRMs) that belongs to the CUGBP family. This protein family is conserved within the animal kingdoms and is composed of six paralogs in mammals, three paralogs known as Bruno or Arrest in *Drosophila melanogaster*, and two paralogs named as ETR-1 and UNC-75 in *Caenorhabditis elegans* [8]. Recently, these different nomenclatures among organisms are unified into CUG binding protein, ELAV-like Family member (CELF) [8]. This protein family is primarily divided into two subfamilies, CELF1-2 and CELF3-6, based on the unique linker sequence between RRM2 and RRM3 [9]. CELF family controls gene expression at a post-transcriptional level by binding not only to the CUG repeat element [10] but also to the GU-rich element (GRE) in the target mRNA [11–13]. In humans, CELF1 activates and inhibits the p21 and p27 expression by binding to the 5’ UTRs of those mRNAs, respectively [14, 15]. In invertebrates, there has been no report of CELF ortholog function via the 5’ UTRs of the target mRNAs. In this study, we renamed *D*. *magna* CUGBP1 ortholog as CELF1 and analyzed its function in *Dsx1* regulation. Our results demonstrate that the CELF1 suppresses the *Dsx1* expression via its 5’ UTR.

## Results

### Sequence conservation of *D*. *magna* CELF1 ortholog

**Fig 1:**
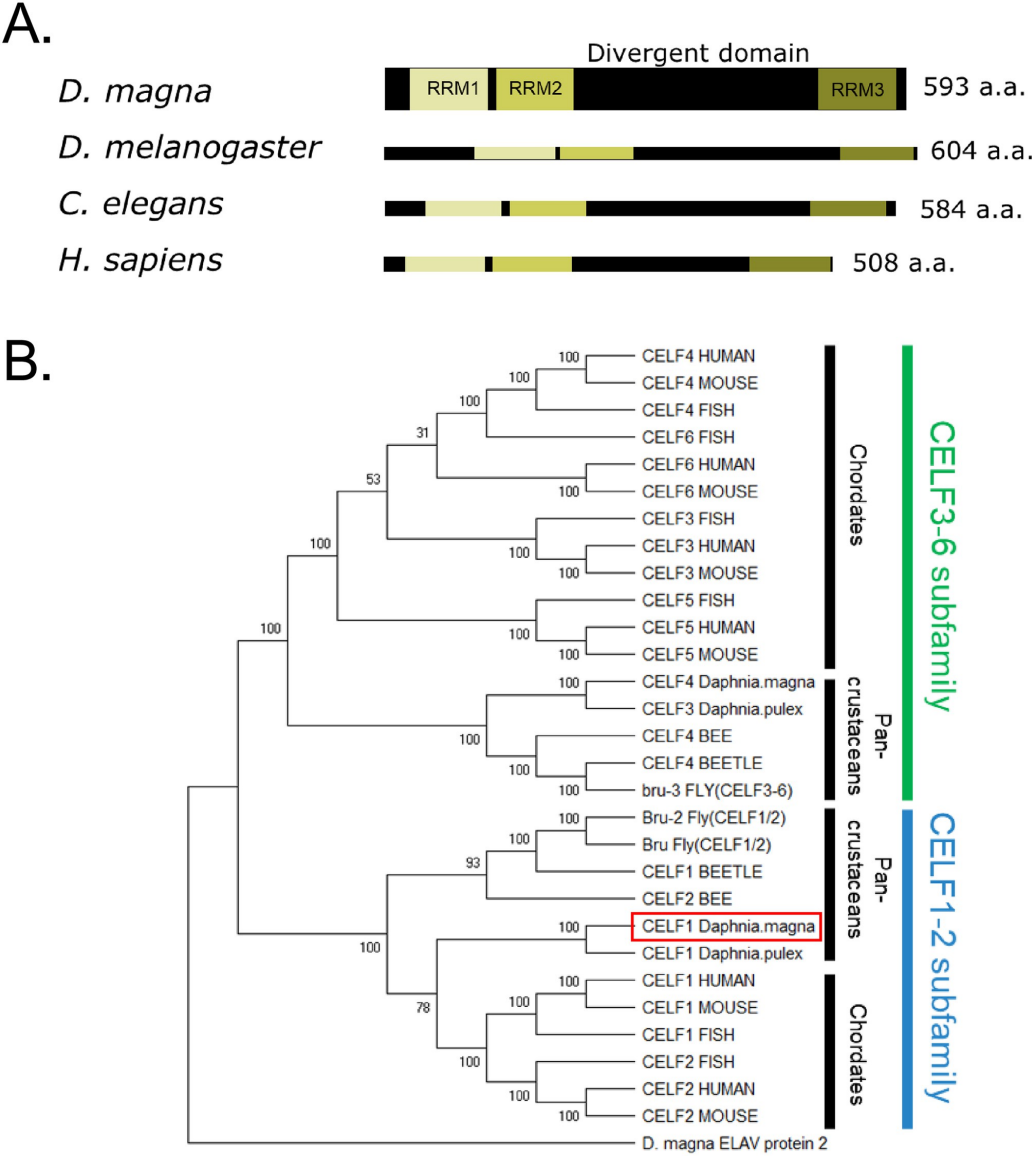
Structure and amino acid sequence conservation of CUGBP Elav-like Family 1 (CELF1) in *D*. *magna*. **(A)** Three conserved RNA Recognition motifs (RRMs) of CELF1 in various species. A unique sequence of the divergent domain is located between RRM2 and RRM3. **(B)** Phylogenetic tree of CELF family protein separates species group; Red square: *D*. *magna* CELF1. The bootstrap values of 1000 replicates were shown next to the branches. The bar indicates branch length and corresponds to the mean number of the differences (P<0.05) per residue along each branch. Evolutionary distances were computed using the p-distance method.

We previously identified an ortholog of human CUG binding protein 1 (CUGBP1) as a candidate protein that binds to *Dsx1α* 5’ UTR [7]. In this study, we renamed *D*. *magna* CUGBP1 ortholog as CELF1. *D*. *magna* CELF1 consists of 593 amino acid residues and harbors three RNA recognition motifs (Fig 1A, S1–S3 Figs). We analyzed the phylogenetic relationship of the *D*. *magna* CELF1 ortholog using amino acid sequences of CELF proteins from various animal species (S1 Table). We confirmed that *D*. *magna* CELF1 belongs to the CELF1-2 subfamily (Fig 1B). Sequence conservation of *D*. *magna* CELF1 suggests that this protein may function as a post-transcriptional regulator.

### *CELF1* expression is not sexually dimorphic during embryogenesis

**Fig 2:**
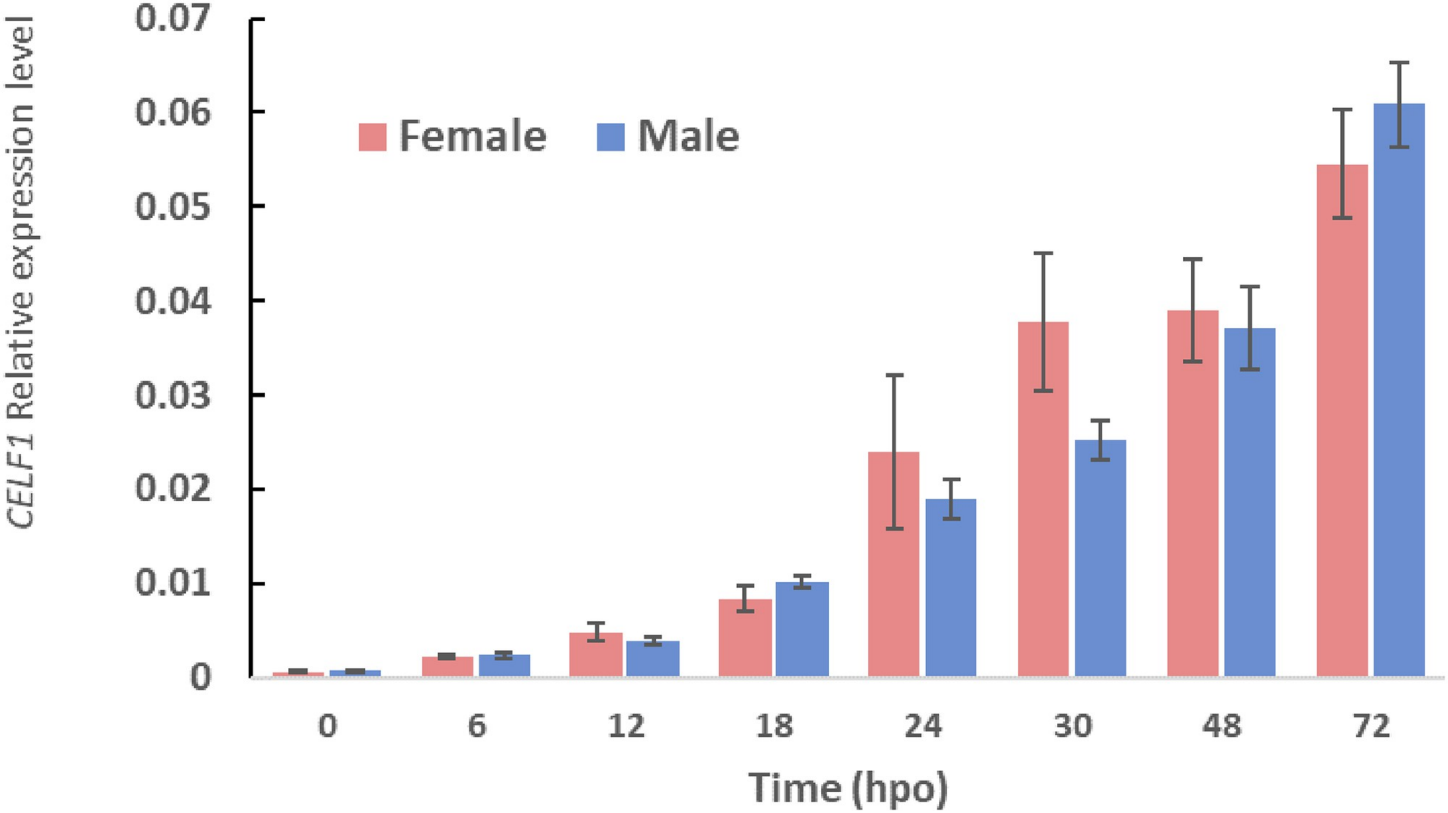
*Daphnia magna* CELF1 temporal expression profile. *D*. *magna CELF1* expression level in embryonic developmental stages. Results were shown in relative expression levels normalized with the ribosomal protein *L32*. hpo, hours post-ovulation. Error bars indicate the standard error of the mean, and the Student’s T-test between both sexes shows no significant difference. N = 3.

The potential association between CELF1 and the *Dsx1α* 5’ UTR suggests that CELF1 might be involved in regulating *Dsx1* expression. Since the sexual regulators often show sex-biased gene expression, we investigated the sexual differences in the temporal expression of *D*. *magna CELF1* at several embryonic developmental stages. The expression level of *CELF1* increased following the embryogenesis stages. However, *CELF1* expression did not show sexual dimorphism at any time points during embryogenesis (Fig 2), which suggests CELF1 functions both in males and females.

### *CELF1* silencing enhanced the *Dsx1* expression in *D*. *magna* embryos

To investigate the role of the *CELF1* ortholog in *D*. *magna*, we silenced *CELF1* expression via RNA interference (RNAi) [16]. First, we injected 300 μM of *CELF1*-specific siRNA (siCELF1) into female eggs from the wild-type of *D*. *magna*. Of the ten injected eggs, 90% (9/10) stopped development before 48 hours post-ovulation, a timing when clear sexual dimorphism in Dsx1 expression and organ formation appears. On the other hand, injection of 100 μM siCELF1 decreased the ratio of the non-viable samples down to 25% (9/36). Based on this result, we decided to use 100 μM siCELF1 to investigate the CELF1 function on the sexual development of *D*. *magna*. To examine the role of *CELF1* in *Dsx1* regulation, we used the *Dsx1* reporter strain (also named Line-B) [17]. This transgenic line has the *mCherry* gene inserted at the position of the *Dsx1* start codon in one allele resulting in a mCherry expression under the control of endogenous *Dsx1* promotor/enhancer. Line-B develops male-specific traits similar to the wild-type because another *Dsx1* allele is intact. This transgenic line also harbors the *H2B-eGFP* gene under the control of the *elongation factor 1α-1* (*EF1α-1*) promotor/enhancer. It allows us to visualize the localization of each cell and map the internal structure of *D*. *magna* [18].

**Fig 3:**
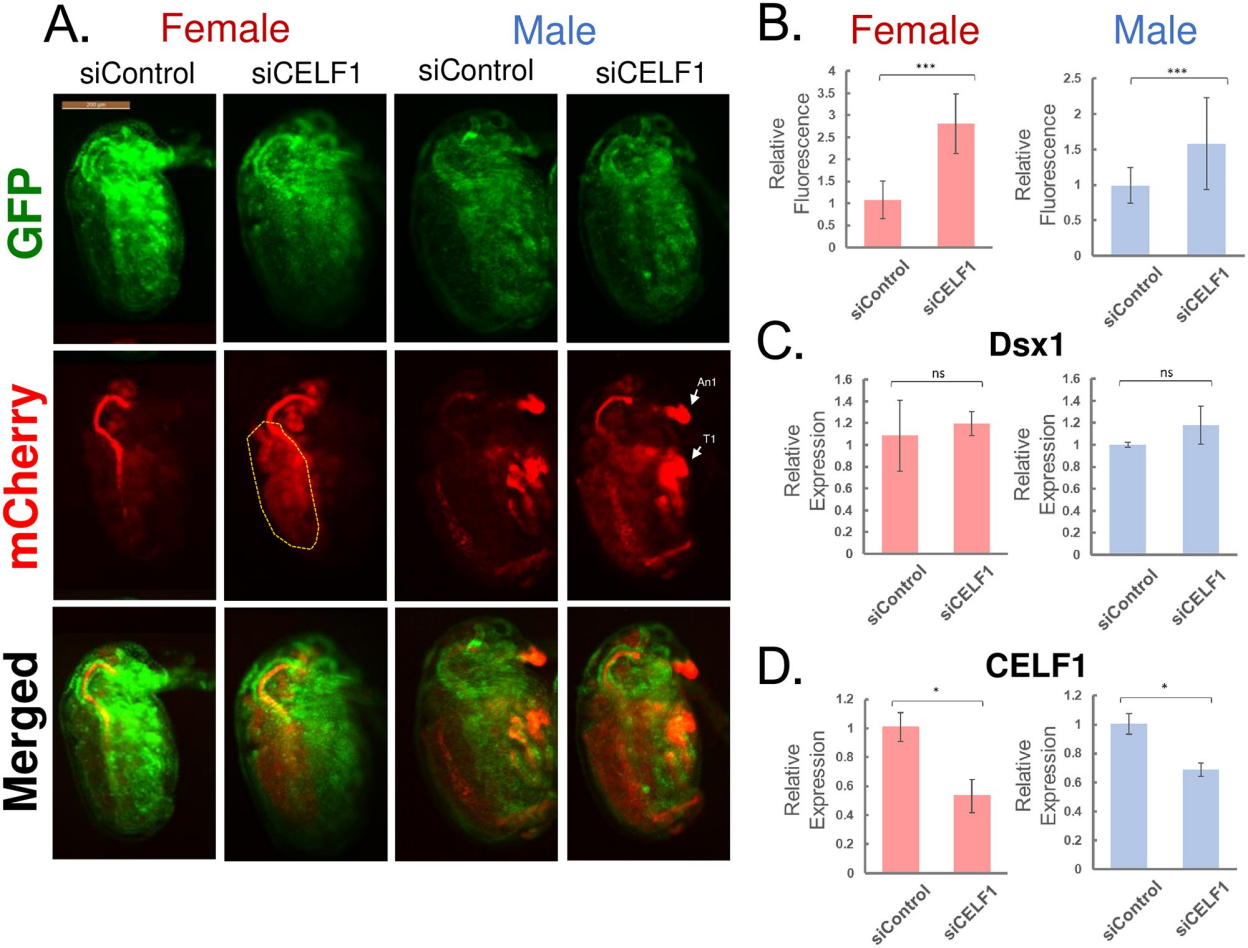
*CELF1* loss of function analysis by RNAi. **(A)** Lateral view of female and male embryos of *Dsx1* reporter strain injected with siCELF1 and siControl observed 48 h post-injection. mCherry fluorescence mirroring *Dsx1* expression and GFP fluorescence allows internal structure mapping. The merged image helps to visualize mCherry expression localization. An1: first antennae, T1: first thoracic appendage, yellow dashed lines: yolk region. **(B)** Relative mCherry fluorescence of siControl and siCELF1 injected samples in female (red) and male (blue) embryos. Error bars indicate the standard error of the mean. N = 12 **(C-D)** Gene transcript level of siCELF1 and siControl injected samples in female (red) and male (blue) 48h post-injection for **(C)** *Dsx1* and **(D)** *CELF1*. RT-qPCR results were shown as relative expression levels to control normalized with the expression level of the ribosomal protein *L32*. Error bars indicate the standard error of the mean. *p<0.05, ***p<0.001, ns: not significant (Student’s T-test). N = 3.

In female embryos, *CELF1* downregulation led to mCherry fluorescence except for the sexually dimorphic traits (Fig 3A and 3B, female) and did not induce sex reversal. The mCherry fluorescence was most visible in the yolk region of female embryos (Fig 3A-yellow dashed line). To investigate the effects of *CELF1* downregulation in male embryos, we collected eggs committed to males by exposing the Line-B mother to fenoxycarb during a critical stage of oocyte development [5, 19]. siCELF1 injection into male eggs increased mCherry fluorescence up to 1.5-fold ubiquitously (Fig 3A and 3B, male). Increased mCherry expression was observed in the whole body, including male-specific tissues (Fig 3A, male), such as the first antennae and the first thoracic appendage [31].

To investigate whether silencing of the *CELF1* changed the *Dsx1* mRNA level or not, we examined the *Dsx1* expression in the siCELF1-injected embryos by the RT-qPCR. siCELF1 injection reduced the target *CELF1* mRNA level (Fig 3D). However, in contrast to the mCherry fluorescence (Fig 3B), the *Dsx1* transcript levels showed no significant difference between *CELF1* RNAi and control embryos (Fig 3C). This result suggests a possibility of *Dsx1* post-transcription regulation by CELF1.

### Overexpression of *CELF1* suppressed *Dsx1* expression in *D*. *magna* male embryos

**Fig 4:**
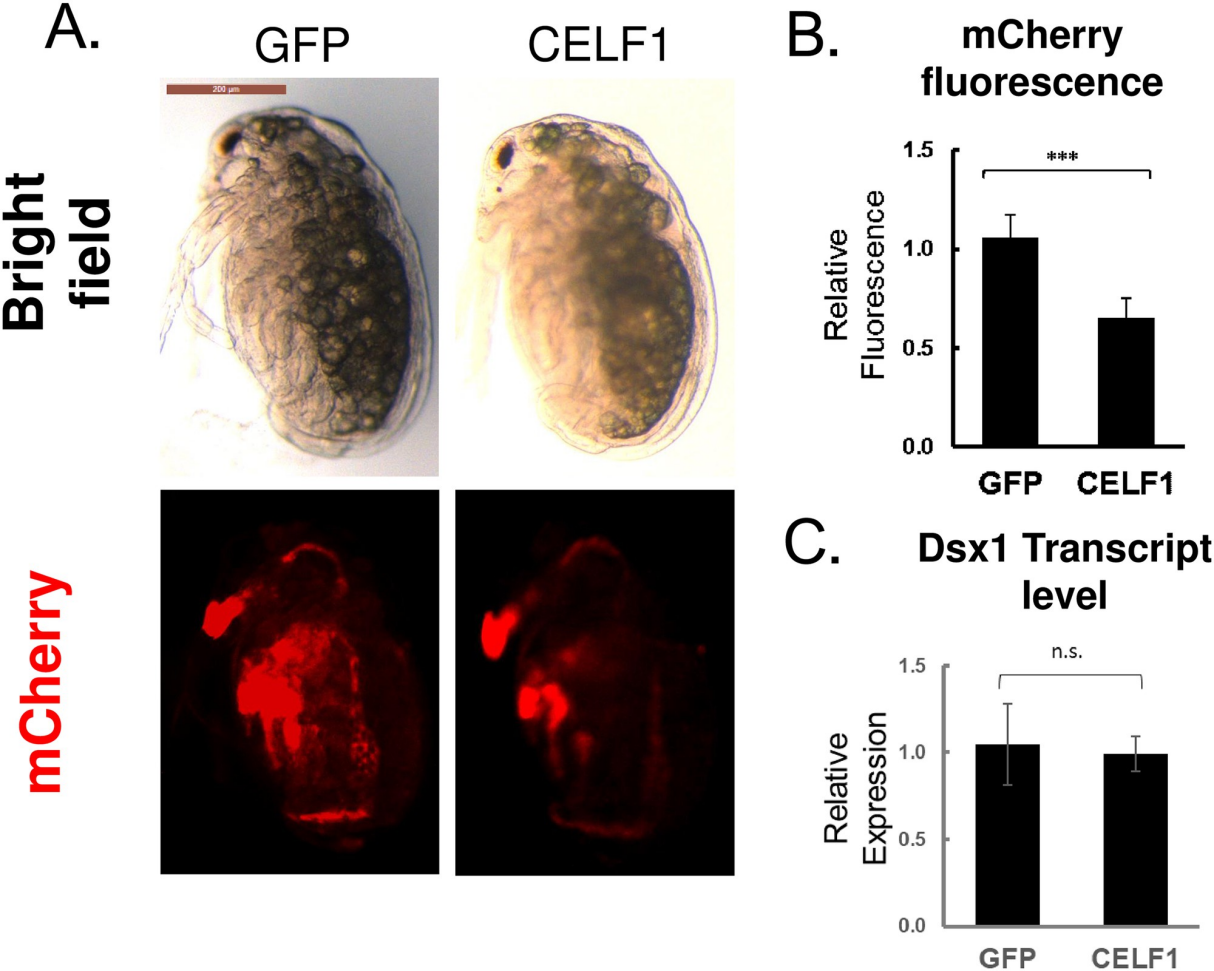
*D*. *magna CELF1* suppressed *Dsx1* expression in male embryos. **(A)** Female and male embryos of *Dsx1* reporter strain variant without H2B-GFP expression injected with any of *CELF1* and *GFP* mRNA, observed at 48 h (lateral view) post-injection. mCherry fluorescence mirroring *Dsx1* expression and Bright field images helps understand the localization of mCherry expression. An1: first antennae, T1: first thoracic appendage. (**B)** Relative mCherry fluorescence of male embryos injected with any of *CELF1* mRNA and *GFP* mRNA at 48 h post-injection. Error bars indicate the standard error of the mean. N = 12 **(C)** *Dsx1* transcript level of male embryos injected with any of *CELF1* mRNA and *GFP* mRNA at 48 h post-injection. RT-qPCR results were shown as relative expression levels to control normalized with the expression of the ribosomal protein *L32*. Error bars indicate the standard error of the mean. ***p<0.001, ns: not significant (Student’s T-test). N = 3.

To further investigate the suppression activity of CELF1 in *Dsx1* expression, we injected in vitro transcribed *CELF1* mRNA into Line-B eggs destined to develop into males. The mCherry fluorescence recapitulating *Dsx1* expression was reduced significantly in the whole body (Fig 4A). mCherry fluorescence intensity was significantly reduced to 0.6-fold in *CELF1* mRNA-injected embryos compared to *GFP* mRNA-injected embryos at 48 h post-injection (Fig 4B). No visible defect was observed at 48 h after CELF1 mRNA injection. In contrast to the significant change of mCherry fluorescence intensity by CELF1 overexpression, there were no significant differences in *Dsx1* transcript levels between *CELF1* mRNA and *GFP* mRNA-injected embryos (Fig 4C).

### CELF1 repressed *Dsx1* expression via the GU-rich element of the *Dsx1α* 5’ UTR in embryos

**Fig 5:**
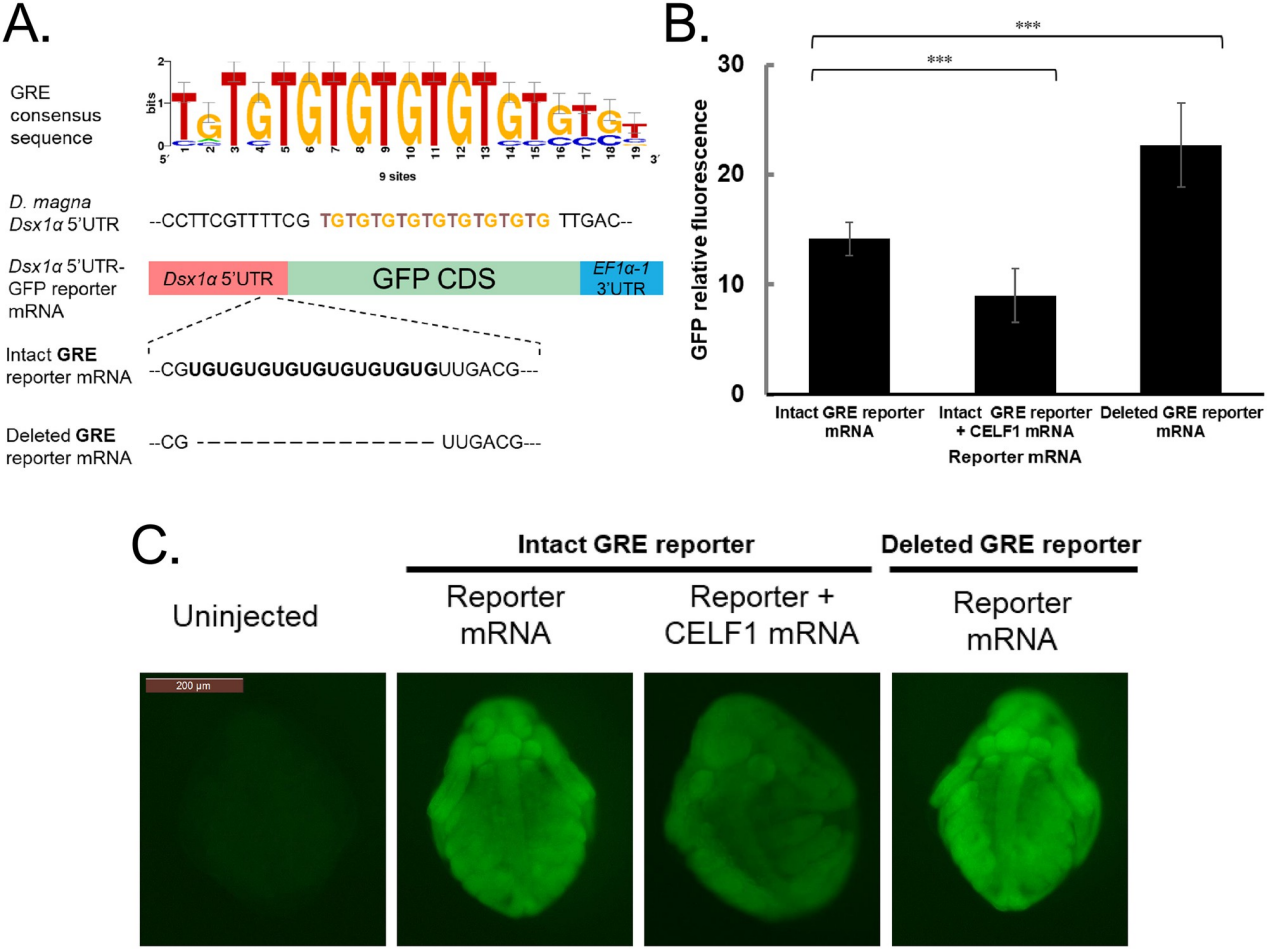
Potential CELF1 binding to *Dsx1*α 5’ UTR through GU-rich Element (GRE). **(A)** GRE consensus sequences and mutated *Dsx1α* 5’ UTR construct with 18 bp deletion of potential CELF1 binding site. **(B)** Relative GFP fluorescence increase of GFP reporter mRNA injected samples in female embryos at 24h post-injection. Error bars indicate the standard error of the mean. ***p<0.001 (Student’s T-test). N = 12. **(C)** Female embryos of wild-type *D*. *magna* injected with GFP reporter mRNA with or without mutated *Dsx1α* 5’ UTR were observed at 24h (ventral view). In addition, the translation efficiency of GFP reporter mRNA was also observed in the presence of *D*. *magna CELF1* overexpression. GFP fluorescence mirroring the ectopic *Dsx1* translation.

We investigated if a potential binding site of CELF1 exists in *Dsx1α* 5’ UTR of *D*. *magna*. Four CELF1 binding sites from *C*. *elegans*, *D*. *melanogaster*, and *Gallus gallus* were used to search for an over-represented CELF1 binding motif (S2 Table). We found that the (UG)_9_ motif was over-represented in all four sequences and *Dsx1α* 5’ UTR (Fig 5A). The previous studies also reported GU-rich Element (GRE) as the preferred binding site of CELF1/2 [11, 12].

To examine whether GRE in the *Dsx1α* 5’ UTR was involved in CELF1-dependent repression of the *Dsx1* expression, we injected *GFP* mRNA harboring *Dsx1α* 5’ UTR with or without the GRE into wild-type female embryos (Fig 5A, intact GRE reporter and deleted GRE reporter). We found that the deleted GRE reporter led to a higher intensity of GFP signal up to 1.5-fold compared to the intact GRE reporter. When we coinjected the intact GRE reporter mRNA with the *CELF1* mRNA, the GFP signal was decreased to 0.5-fold compared to samples without *CELF1* overexpression (Fig 5B and 5C). These results suggest that CELF1 possibly controls *Dsx1* expression via the GRE.

## Discussion

The crustacean *D*. *magna* lacks sex chromosomes and utilizes environmental cues for sex determination. *D*. *magna* produces only female offspring in favorable conditions. In contrast, environmental cues stimulate male production [1, 2]. Previous studies revealed that environmental cues are converted into sesquiterpenoid signaling and activate the Dsx1 gene [3, 4]. This gene codes for the DM-domain transcription factor and orchestrates the male-developmental program [5]. Manipulation of Dsx1 expression and activity in females leads to the generation of intersex phenotype [5], demonstrating that the *Dsx1* gene must be tightly silenced in females throughout development, and it would be upregulated precisely in a spatio-temporal manner for male production. Thus, unraveling the regulatory mechanism of Dsx1 expression is essential for understanding how sex is determined in this species. In this study, we investigated the function of the RNA binding protein CELF1, which was identified as one of the proteins associated with the 5’ UTR of the *Dsx1α* isoform [7].

CELF1 has been reported to target sex-determining and development genes across many species. First, as an alternative splicing factor, CELF1 works synergistically with Lark protein orthologs to promote male-specific splicing of *B*. *mori Dsx* [20]. Second, as an mRNA destabilizing factor, it works antagonistically with *Ol-bsf* to reduce Medaka fish sex-determining gene *dmrt1bY* expression and hinder male gonad development [21]. Third, as a translational repressor, CELF1 (Bru) represses the master sex-determining gene *Sxl* in *Drosophila*, promoting the male dosage compensation and somatic differentiation cascade [22, 23]. In addition, CELF1 has a critical role in controlling gonadal development in the fruit fly, mice, and nematode [24–27]. During evolution, CELF1 might be repeatedly used in the animal sex-determining pathways.

We found that CELF1 functions as a post-transcriptional repressor of the *D*. *magna Dsx1* gene. Our result showed that CELF1 repressed *Dsx1* expression possibly via binding to the GU-rich element (GRE) of the *Dsx1α* 5’ UTR. GRE is known to be a predominant binding site of CELF1 [11–13]. This protein binds to the 5’ UTR of *p21* and *C/EBPβ* mRNA in human cancer cells, enhancing their translation efficiency [28, 29]. On the contrary, CELF1 functions as a negative translation regulator via binding to the 5’ UTR of *p21* mRNA in the mouse lens cell line [14] and *p27* mRNA in the breast cancer cell [21]. Since (1) Dsx1 transcript level did not change by CELF1 silencing and overexpression and (2) deletion of GRE from the *Dsx1α* 5’ UTR increased its GFP reporter expression, it may be possible that CELF1 may suppress Dsx1 translation via its 5’ UTR.

During embryogenesis, CELF expression did not show any sexual dimorphism. In addition, in both sexes, CELF1 silencing de-repressed Dsx1 expression. The *Dsx1* expression increase only in male 6 hours after ovulation and is localized in the sexually dimorphic organs such as the first antennae, first thoracic appendage, and gonads [5]. In the knockdown females, *Dsx1* expression was observed in the yolk region and was not detected in the sexually dimorphic traits such as the first antennae suggesting CELF1 is important but insufficient. This result may explain the absence of sex reversal in females. Further loss-of-function experiment to observe the sex reversal was not possible due to CELF1 affecting the embryo’s viability. Overexpression of CELF1 in male embryos reduced mCherry fluorescence, and the importance of the GRE element for repression of *Dsx1α* expression was successfully evaluated in female embryos. Based on these data, CELF1 possibly has the ability to repress Dsx1 expression both in females and males.

The non-sex-specific role of CELF1 on Dsx1 repression could provide insight into the CELF1 function to set the threshold of Dsx1 expression. In females, Dsx1 protein from noisy expression may bind to the potential Dsx1 binding site upstream of the *Dsx1α* transcription start site [6] and self-activate its expression via a positive feedback loop. CELF1 might avoid unintended Dsx1 translation and subsequently eliminates the generation of sexual ambiguity in females. Since Dsx1 protein is expressed in a tissue and time-specific manner in males [17], it would be possible that CELF1 contributes precise control of Dsx1 activation in males. In this model, we must include the RNA binding protein Shep and long noncoding RNA (lncRNA) named DAPALR [7, 30]. Shep also represses Dsx1 translation by binding to the *Dsx1α* 5’ UTR, and DAPALR is an endogenous competing RNA that sequestered Shep. Further studies to prove this model are needed in the future, which in turn may allow us to recognize an elegant control of sex determination in *D*. *magna*.

## Conclusion

Our results demonstrate the molecular function of CELF1 in the repression of the male-determining gene Dsx1 expression in vivo. This function may contribute to avoiding sexual ambiguity in females and achieving spatio-temporal expression in males. We anticipate that this work will be a basis for understanding the regulatory mechanism of *Dsx1* expression in *D*. *magna* with the environmental sex-determination system.

## Materials and methods

### *Daphnia magna* stains and transgenic lines cultures

The wild-type (WT) and the transgenic lines used in this study share the same genetic background (NIES clone). They were cultured in AdaM medium [31] as previously described [5]. The RNAi experiment used a *Dsx1*-reporter strain with the mCherry ORF introduced upstream of the *Dsx1* coding sequence [17]. This line also has eGFP fused to histone H2B gene under the control of the *elongation factor 1α-1* promotor/enhancer. Line B minus, a variant of this strain without H2B-eGFP expression [7], was also used for the ectopic expression experiment. Male *Daphnia* was obtained by exposing female *Daphnia* (2-3- weeks old) to 1 μg/L of the synthetic juvenile hormone analog, Fenoxycarb (Wako Pure Chemical, Osaka, Japan) [5].

### Phylogenetic analysis of the *D*. *magna CELF1* gene

Amino acid sequences of CELF family proteins were obtained from the NCBI database (http://www.ncbi.nlm.nih.gov/) as shown in S1 Table. Each protein’s whole amino acid or RRMs sequences were subjected to multiple sequence alignment and used to construct the phylogenetic tree. Based on the amino acid sequences, multiple sequence alignments were constructed using the Clustal W in MEGA version 10.0.5 [32]. The following settings were used for the analysis: pairwise alignment parameters: gap opening penalty = 10.00, gap extension penalty = 0.1, and identity protein weight; matrix multiple alignment parameters: gap opening penalty = 10.00, gap extension penalty = 0.20, and delay divergent cut-off = 30%. The phylogenetic reconstruction was performed using the p-distance algorithm and the neighbor-joining method implemented in MEGA.

### Microinjection

Foreign material injection into *Daphnia* eggs was performed using an established protocol [16]. Freshly ovulated eggs from 2–3 weeks old *Daphnia* mothers were obtained using microdissection and transferred into an ice-chilled M4 medium [33] containing 80 mM sucrose (M4-sucrose). For the injection solution, 2 mM Lucifer Yellow (Invitrogen, Carlsbad CA, USA) was mixed as an injection marker for each experiment. Following 1 hour after microinjection, survived eggs were transferred into each well of 96-well plates with 100 μL of M4-sucrose medium and were kept in an incubator at 23°C.

### *CELF1* RNAi in embryos

Small interference RNAs were designed using the website Block-iT RNAi Designer at https://rnaidesigner.thermofisher.com/rnaiexpress/. The sequence of this siRNA is as follows: siCELF1 (5’- GCAATGAGCGTAAACTCTT -3’). As a negative control, siRNA targeting a random sequence that does not affect the *Daphnia* development was used: siControl (5’- GGUUAAGCCGCCUCACAUTT-3’) [34]. The siRNA oligonucleotides were dissolved in DNase/RNase-free water (Life Technologies Inc.; Grand Island, NY, USA). Two nucleotides dTdT were added to each 3′ end of the siRNAs. The siRNAs were diluted with the injection marker 2 mM Lucifer Yellow dye (Invitrogen, Carlsbad CA, USA) to have the final concentration of 100 μM or 300 μM. The injection cocktails were injected into female or male eggs of the *Dsx1*-reporter strain *Daphnia*. Samples were then observed at 24 h after injection and collected at 48 h for RNA extraction and cDNA synthesis as previously described [30].

### *CELF1* overexpression in embryos

To create chimeric CELF1 mRNA, CELF1 CDS was amplified by PCR using synthesized cDNA derived from NIES strain RNA extraction. Then, CELF1 CDS was subcloned into a pCS2 vector harboring the T7 polymerase promoter, *EF1α1* 5′ UTR, and 3’ UTR derived from the chimeric DsRed2 mRNA expression plasmid construct [35] using GeneArt Seamless Cloning and Assembly Enzyme Mix (Invitrogen, Carlsbad CA, USA). The plasmid construct was named pEF1α1-CELF1. For control mRNA preparation, the *CELF1* CDS of pEF1α1-CELF1was then replaced with the CDS of *GFP* using seamless cloning. The GFP region was amplified from the 4xEcRE-H2B-GFP plasmid [36].

To prepare the GFP reporter mRNA harboring *Dsx1α* 5′ UTR, pEX-*Dsx1* 5′ UTR::GFP [7] was used as a template for mRNA synthesis. This plasmid was used as a template to delete the potential *CELF1* binding site using the primer set as follows: Forward (5’- TCCCCTTCGTTTTCGTTGACGTTTTCATTTCCA-3’) and Reverse (5’- AAATGAAAACGTCAACGAAAACGAAGGGGAAAT-3’) resulting in the generation of pEX-*Dsx1* 5′ UTR GRE mutant::GFP. For internal control, the *CELF1* CDS of pEF1α1-CELF1 was then replaced with the CDS of *mCherry* using seamless cloning to produce pRCS21-EF1α-1-mCherry, which was used to synthesize EF1α-1-mCherry mRNA as a template. The *mCherry* region was amplified from the bicistronic reporter plasmid in the previous study [37].

*In vitro* transcription and poly(A) tail addition were performed with mMESSAGE mMACHINE T7 RNA Polymerase and Poly(A) Tailing kits, respectively (both Ambion, Foster City, CA, USA). The synthesized RNA size and the attached poly(A) tail length were analyzed by denaturing formaldehyde gel electrophoresis.

CELF1 ectopic expression was performed by injecting 3200 ng/ul CELF1 mRNA mixed with 2 mM Luciferase Yellow dye into Line-B variant eggs without endogenous GFP fluorescence called Line-B minus [7]. These eggs were induced to be male before injection, as mentioned above. The sample was observed under a fluorescent microscope to measure the mCherry fluorescence and collected for total RNA extraction at 48 h. For GFP reporter assay, 40 ng/ul *Dsx1* 5′ UTR::GFP reporter mRNA with or without GRE was coinjected with 40 ng/ul mCherry mRNA into wild-type female eggs. The sample was observed under a fluorescent microscope to measure GFP fluorescence at 24 h.

### Total RNA extraction and cDNA synthesis

Total RNA was extracted using Sepasol-RNAI solution (Nacalai Tesque; Kyoto, Japan) according to the manufacturer’s protocol and followed by phenol/chloroform purification. The purified total RNA was subjected to cDNA synthesis using random primers (Invitrogen; Carlsbad, CA, USA) and the SuperScriptIII Reverse Transcriptase (Invitrogen) according to the manufacturer’s recommended protocol.

### Quantitation of the fluorescence

Injected sample photos were taken using Leica DC500 CCD Digital Camera mounted on a Leica M165FC fluorescence microscope (Leica Microsystem, Mannheim, Germany). Fluorescence photography was done using GFP2 and mCherry filters under the following conditions: 1.0 s exposure time, 3.0x gain, 1.0 saturation, and 1.0 gamma for GFP and 2.0 s exposure time, 8.0x gain, 1.0 saturation, and 1.6 gammas for mCherry. The fluorescence intensities were calculated using ImageJ software, following the calculation protocol from a previous study [35]. The measurement of background fluorescence normalized the total embryo fluorescence of each sample. Relative Fluorescence Intensity (RFI) was calculated following the protocol of a previous study [7].

### Quantitative RT-PCR

The temporal changes in *CELF1* expression level during embryogenesis were analyzed using previously synthesized cDNA [6] from male and female *Daphnia* at different time points (0, 6, 12, 18, 24, 30, 48, and 72 h after ovulation). Each cDNA was subjected to RT-qPCR using CELF1 specific primer set as follows: Forward (5’- CGGCATCCAGCAATTCACTAC-3’) and Reverse (5’- CGTCACACTTCCACCACCAC-3’). To check the expression level changes of the genes of interest (*CELF1* and *Dsx1*) between the siControl- and siCELF1-injected samples or GFP mRNA- and CELF1 mRNA-injected samples, cDNA from 48-hour embryos were subjected to RT-qPCR. The expression level of *CELF1* and *Dsx1* in RNAi or overexpression experiments were prepared as three replicates for RT-qPCR. mRNA transcripts were measured using StepOnePlus^™^ Real-Time PCR System (Agilent Technologies), Power SYBR Green qPCR Mastermix (Invitrogen, Carlsbad CA, USA), and a specific primer designed to amplify <150 bp PCR products under the following conditions: 95°C for 10 min, 40 cycles of 95°C for 15 sec and 60°C for 1 min, and last amplification round of 95°C for 1 min, 55°C for 30 sec, and 95°C for 30 sec. *Dsx*1 specific primer set sequence were designed as follow: Forward (5’- AAGTTTGGTGTAGGGGAGGATGAG -3’) and Reverse (5’- CCATTCATCATTACCAAATCCCTTC -3’). Expressions based on the Ct value during amplification were calculated and normalized by quantitating the expression level of ribosomal protein gene *L32* [38]. Finally, dissociation curve analysis and gel electrophoresis were performed to confirm the correct amplicon size and the absence of non-specific bands.

## Supporting information

S1 Fig Multiple sequence alignments of CELF1 RRM1 orthologs. Conserved amino residues with identical or similar characteristics were colored by the ClustalX color scheme. Dashes indicate gaps in the alignment. Numbers represent amino acid positions.
(TIF)

S2 Fig Multiple sequence alignments of CELF1 RRM2 orthologs. Conserved amino residues with identical or similar characteristics were colored by the ClustalX color scheme. Dashes indicate gaps in the alignment. Numbers represent amino acid positions.
(TIF)

S3 Fig Multiple sequence alignments of CELF1 RRM3 orthologs. Conserved amino residues with identical or similar characteristics were colored by the ClustalX color scheme. Dashes indicate gaps in the alignment. Numbers represent amino acid positions.
(TIF)

S1 Table Nomenclature and CELF family orthologs. (DOCX)

S2 Table Target sequences of CELF1 orthologs. (DOCX)
