# Supplementary figures and images for: CELF1 represses Doublesex1 expression via its 5’ UTR in the crustacean Daphnia magna
Source: PLoS One. 2022 Oct 14;17(10):e0275526. doi: 10.1371/journal.pone.0275526 (PMC9565731; doi:10.1371/journal.pone.0275526)

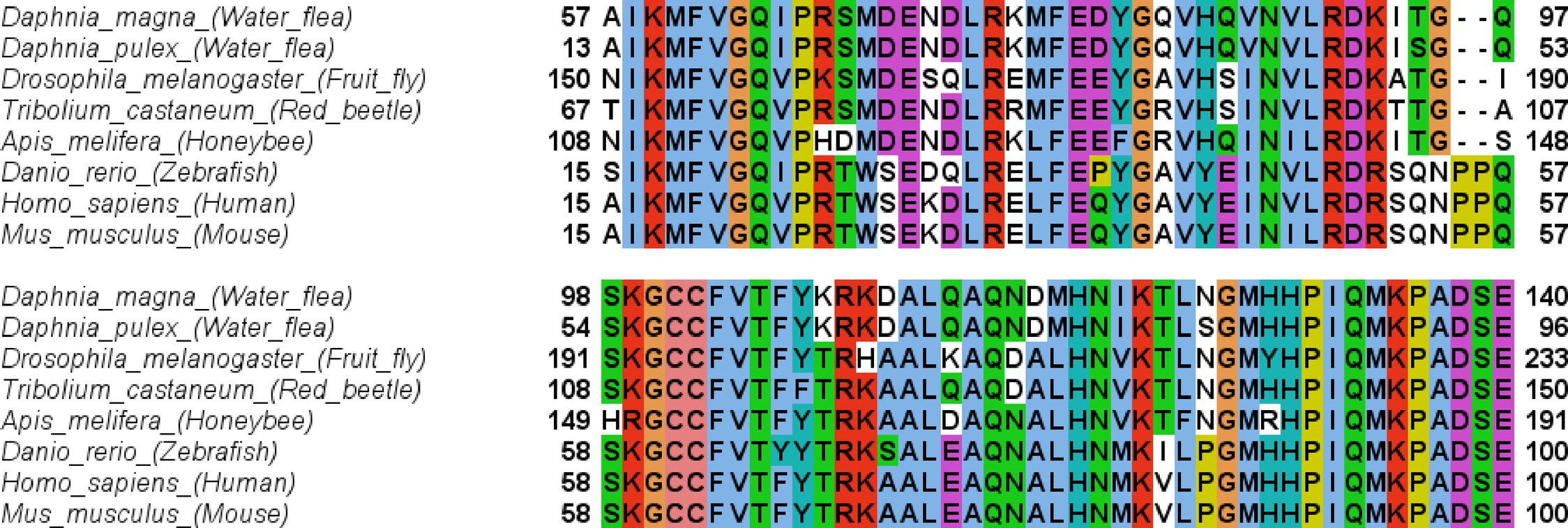

Supplement: S1 Fig — Conserved amino residues with identical or similar characteristics were colored by the ClustalX color scheme. Dashes indicate gaps in the alignment. Numbers represent amino acid positions. (TIF) [file pone.0275526.s001.tif]

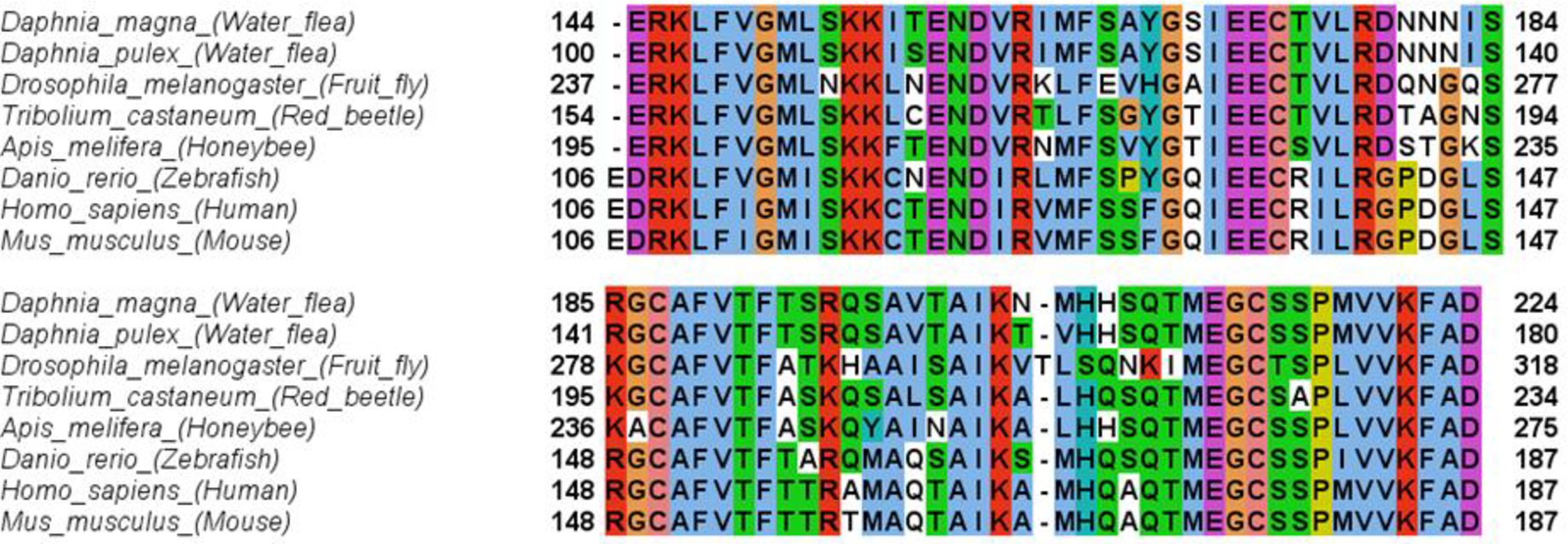

Supplement: S2 Fig — Conserved amino residues with identical or similar characteristics were colored by the ClustalX color scheme. Dashes indicate gaps in the alignment. Numbers represent amino acid positions. (TIF) [file pone.0275526.s002.tif]

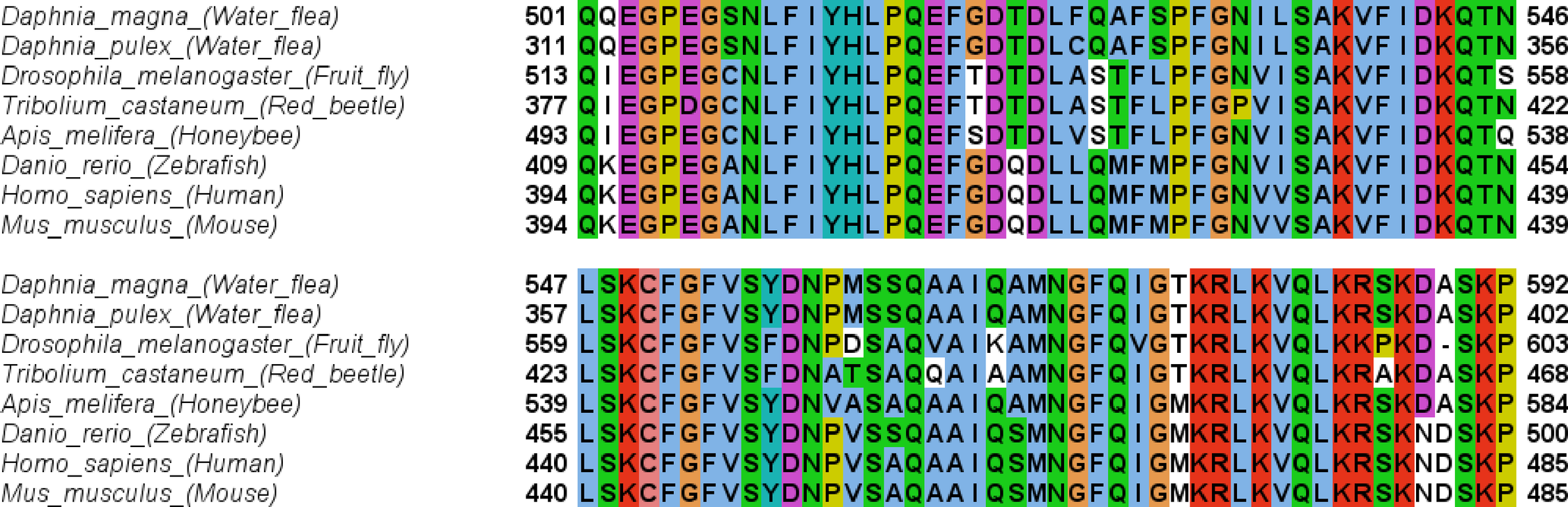

Supplement: S3 Fig — Conserved amino residues with identical or similar characteristics were colored by the ClustalX color scheme. Dashes indicate gaps in the alignment. Numbers represent amino acid positions. (TIF) [file pone.0275526.s003.tif]
