# Supplementary material for: CELF1 represses Doublesex1 expression via its 5’ UTR in the crustacean Daphnia magna
Source: PLoS One. 2022 Oct 14;17(10):e0275526. doi: 10.1371/journal.pone.0275526 (PMC9565731; doi:10.1371/journal.pone.0275526)
Supplement: Supplementary file 2 [file pone.0275526.s004.docx]

| Species | CELF family member(s) | Gene | Uniprot Accession number |
|---|---|---|---|
| *H. sapiens* Human | CELF1 | CELF1 | Q92879 |
| | CELF2 | CELF2 | O95319 |
| | CELF3 | CELF3 | Q5SZQ8 |
| | CELF4 | CELF4 | Q9BZC1 |
| | CELF5 | CELF5 | Q8N6W0 |
| | CELF6 | CELF6 | Q96J87 |
| *M. musculus* Mouse | CELF1 | *celf1* | P28659 |
| | CELF2 | *celf2* | Q9Z0H4 |
| | CELF3 | *celf3* | Q8CIN6 |
| | CELF4 | *celf4* | Q7TSY6 |
| | CELF5 | *celf5* | A0A5F8MPH2 |
| | CELF6 | *celf6* | Q7TN33 |
| *D. rerio* Zebrafish | CELF1 | *celf1* | Q9IBD0 |
| | CELF2 | *celf2* | Q6P0B1 |
| | CELF3 | *celf3* | Q9IBD1 |
| | CELF4 | *celf4* | Q6DGV1 |
| | CELF5 | *celf5* | B3DJA7 |
| | CELF6 | *celf6* | A0A0R4ID04 |
| *D. melanogaster* Fruit fly | CELF1-2 | *bru* | Q960Z4 |
| | | *bru-2* | Q0E8R3 |
| | CELF3-6 | *bru-3* | Q9VU91 |
| *Outgroup D. magna* | - | ELAV protein 2 | APZ42_028252 |

| Species | CELF family member(s) | Gene | Genebank Accession number |
|---|---|---|---|
| *A. melifera* Honey Bee | CELF1-2 | CELF2* | XP_026294967.1 |
| | CELF3-6 | CELF4* | XP_006557659.1 |
| *T. castaneum* Red Flour Beetle | CELF1-2 | CELF2* | XP_015838769.1 |
| | CELF3-6 | CELF4* | KYB25053.1 |
| *P. promelas* Bonefish | CELF1 | *celf1* | XP_039541988.1 |
| *C. elegans* Roundworm | CELF1-2 | *etr-1* | NP_001367867.1 |
| | CELF3-6 | *unc-75* | NP_492958.3 |
| *D. magna* Water flea | CELF1-2 | CELF1* | XP_032788705 (JAL60823.1) |
| | CELF3-6 | CELF4* | JAM69815.1 |
| *D. pulex* Water flea | CELF1-2 | CELF1* | EFX71524 |
| | CELF3-6 | CELF3* | XP_046453064.1 |

**putative name*
