# Supplementary material for: CELF1 represses Doublesex1 expression via its 5’ UTR in the crustacean Daphnia magna
Source: PLoS One. 2022 Oct 14;17(10):e0275526. doi: 10.1371/journal.pone.0275526 (PMC9565731; doi:10.1371/journal.pone.0275526)
Supplement: Supplementary file 3 [file pone.0275526.s005.docx]

Organism	CELF1 orthologs	Gene target	Sequence	Method	Reference
C. elegans	Etr-1	*nrx-1*	GAGGCGGCTTTTGAATGAAAAAAAACCC	CLIP-Seq	**Chen *et al.* 2016** DOI:10.7554/eLife.16072
		unc-75	TACACATCTGTGTGTGTGTGTGTGTGTGTGTGTCCCCCGCC		
G. gallus	CUGBP/ CELF1	*cTnTMse*	AATAAATCGCGGGTCGGTGTGTCCTGTGCCTTTCCCTGCTTGGGAAA	CLIP	**Ladd *et al.* 2001** DOI:10.1128/MCB.21.4.1285-1296.2001
D. melanogaster	Bruno	*osk*	GATCCAATGTATGTTAATTGTATGTATTA	CLIP	**Webster *et al.* 1997** DOI:10.1101/gad.11.19.2510
